# Bilateral *Trichosporon asahii* keratitis after ptosis correction: A case report

**DOI:** 10.1097/MD.0000000000026688

**Published:** 2021-07-23

**Authors:** Jeongah Shin, Woo Young Son, Kyong Jin Cho, Chang Rae Rho

**Affiliations:** a Department of Ophthalmology, Daejeon St. Mary's Hospital, College of Medicine, The Catholic University of Korea, Daejeon, Republic of Korea; b Department of Ophthalmology, College of Medicine, Dankook University, Cheonan, Republic of Korea.

**Keywords:** cornea, keratitis, *Trichosporon asahii*, voriconazole

## Abstract

**Rationale:**

Fungal keratitis (FK) is a severe vision-threatening disease that can lead to corneal perforation or endophthalmitis despite proper treatment. It is important to diagnose the disease promptly due to its indolent nature and disproportionate disease symptoms. *Trichosporon asahii* is reported rarely as the causative organism of FK. We report a case of highly unusual bilateral *T asahii* keratitis following ptosis surgery.

**Patient concerns:**

An 86-year-old female underwent bilateral levator resection surgery for ptosis. Postoperatively, the patient complained of gradually worsening bilateral ocular pain and a decrease in visual acuity associated with a chronic non-healing epithelial defect.

**Diagnoses:**

Both eyes of the patient were evaluated using best-corrected visual acuity, intraocular pressure, slit-lamp examination, fundus examination, and corneal culture. Multifocal deep stromal infiltrates were found in both corneas. Cultures from both corneal ulcers revealed growth of *T asahii*. Optical coherence tomographic examination showed bilateral macular edema.

**Interventions:**

The patient was treated with revisional ptosis surgery, an antifungal agent for the corneal ulcer, and intravitreal injection of steroid for macular edema.

**Outcomes:**

Both eyes recovered well. Her best-corrected visual acuity improved from 20/200 to 20/40 in the right eye and from 20/100 to 20/40 in the left eye.

**Lessons:**

FK can develop in the cornea when certain risk factors are present, including recent lid surgery, chronic keratitis, and steroid eye drop use. Identification and correction of risk factors can be beneficial in the treatment of FK.

## 1 Introduction

Fungal keratitis (FK) is one of the most difficult corneal infections to diagnose and treat successfully. The reported incidence of infectious FK varies from 6% to 35%.^[1,2]^*Candida* spp., *Aspergillus* spp., and *Fusarium* spp. are most commonly isolated in FK,^[3,4]^ while *Trichosporon asahii* keratitis is relatively rare. A literature search produced a case of *T asahii* infection in a patient with a type I Boston keratoprosthesis who wore a contact lens^[5]^ and in 1 case of 393 isolates at a tertiary care center in north India.^[6]^ Bilateral FK is rare and is mainly reported to be associated with laser in situ keratomileusis.^[7–9]^ We present a highly unusual case of bilateral *T asahii* keratitis in a patient who wore soft contact lenses after ptosis surgery.

## 2 Case presentation

**Figure 1:**
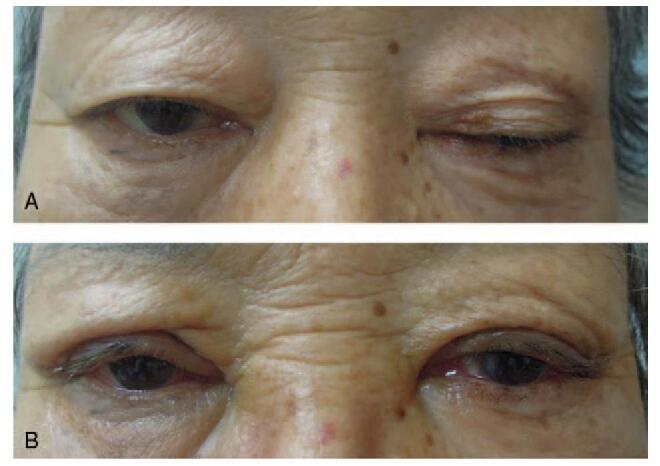
(A) Aponeurotic ptosis was more prominent in the left eye. (B) One week after levator resection.

**Figure 2:**
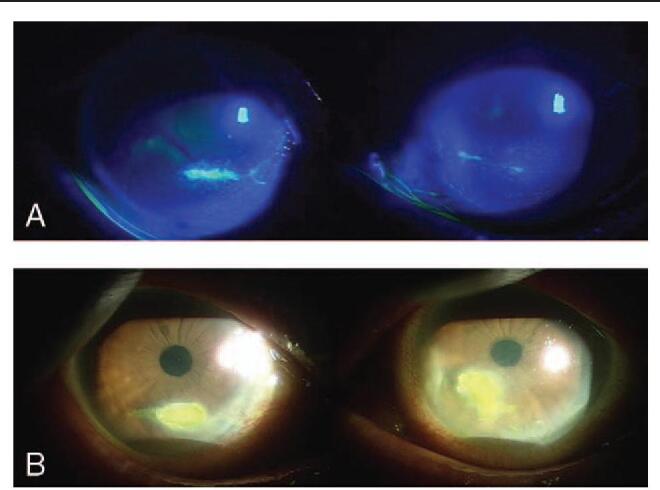
(A) Both corneas showed newly developed, non-healing corneal epithelial defects 2 months after lid surgery. (B) The epithelial defect was aggravated, and the size of the defect increased 4 months after lid surgery.

**Figure 3:**
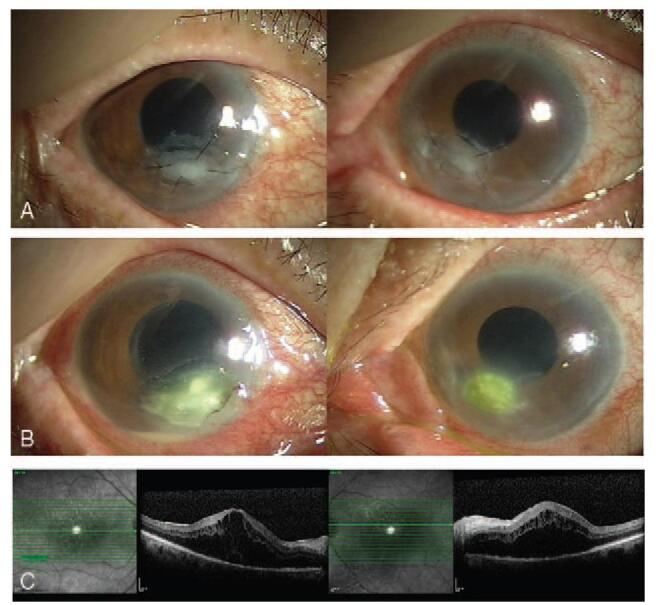
(A) A dry amniotic membrane was transplanted using an epithelial-side-up configuration in both eyes. (B) One week after the amniotic membrane was removed, hypopyon and dense multifocal corneal infiltrates developed. The intraocular pressure increased in both eyes. (C) Severe cystoid macular edema was noted after fungal ulcers developed in both eyes.

**Figure 4:**
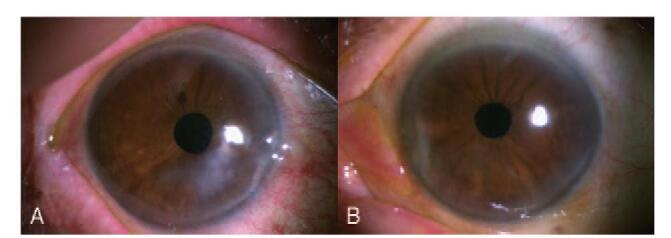
The integrity of the corneas were restored without evidence of epithelial defects. The stromal opacity remained.

We present an unusual case of bilateral FK that arose in an 86-year-old female patient after ptosis surgery. This patient visited our clinic for aponeurotic ptosis, which was more evident in the left eye (Fig. 1A). Her best-corrected visual acuity (BCVA) was 20/20 in both eyes. She denied any systemic medical history, including diabetes or hypertension. An oculoplastic surgeon performed levator resection surgery in both eyelids without complications (Fig. 1B). A persistent, non-healing, corneal epithelial defect was observed in both eyes despite complete closure of both eyelids after surgery (Fig. 2A). The epithelial defect was worse in the right eye and was refractory to conventional treatments, including artificial tears, 20% serum eye drops, and eye patching. Therapeutic contact lenses were prescribed to heal the persistent corneal epithelial defect, but sterile uveitis developed; intraocular pressure (IOP) abruptly increased to >40 mmHg. At 4 months postoperatively, the uveitis and IOP were both controlled. However, the patient's epithelial defects persisted, and the size of the wound increased in both eyes (Fig. 2B). Her BCVA was 20/40 in the right eye and 20/50 in the left eye. To promote healing of the nonresponding epithelial defects, dry amniotic membranes were sutured to the corneal stromal bed using the epithelial-side-up configuration in both eyes (Fig. 3A). The patient was instructed to instill 0.5% moxifloxacin and 0.1% fluorometholone eye drops 4 times a day. After 2 weeks, the membranes still had not attached to the corneal stroma, and the sutured amniotic membranes were removed from the corneas. One week after their removal, a prominent hypopyon was found in her right eye (Fig. 3B), and dense, deep multifocal stromal infiltrates were observed in both eyes. The IOP increased to 43 mmHg in the right eye and 36 mmHg in the left eye with prominent cells and flares in both anterior chambers. The corneal stromal beds of both eyes were scraped using a surgical blade for microbiologic analysis. The culture workup revealed *T asahii* growth in both eyes. The patient was treated with 0.15% amphotericin B eye drops each hour and 100 mg of oral fluconazole daily. Three weeks after removal of the amniotic membrane, she underwent lid revision surgery to release adhesions of the levator muscle in both eyes. The topical antifungal was changed from 0.15% amphotericin B to 1% voriconazole (by diluting the intravenous formulation of voriconazole) eye drops since the clinical response was slow. The corneal ulcers improved, and epithelial healing was complete 1 month after lid revision surgery. In total, the patient took oral fluconazole daily for 6 weeks and was instilled 1% voriconazole hourly for 3 months. During the course of treatment, bilateral cystoid macular edema developed (Fig. 3C). The BCVA was 20/200 in the right eye and 20/100 in the left eye. Macular edema was treated with intravitreal injection of triamcinolone acetonide. At her last visit, which was 3 years after the development of corneal ulcers, the BCVA was 20/40 in both eyes, and both corneas maintained epithelial integrity without any defects. However, prominent paracentral corneal thinning and opacity remained in the right cornea (Fig. 4). This study was approved by the Institutional Review Board of Daejeon St. Mary's Hospital (DC21ZISI0026) and was conducted in accordance with the tenets of the Declaration of Helsinki. Informed consent was obtained from the patient for publication of this case report.

## 3 Discussion

FK is a serious disease that can lead to moderate to severe vision loss despite appropriate treatment. Several characteristic clinical features have been proposed for filamentous FK, which is characterized by serrated margins, raised slough, a dry texture, satellite lesions, and any coloration other than yellow.^[10]^ However, the symptoms of FK are more indolent and can be disproportionate relative to the size of the ulcer compared to those of bacterial keratitis. This situation can complicate and delay FK diagnosis, which can lead to a poor outcome. Corneal perforation in FK is 5 to 6 times more common than in bacterial keratitis and can lead to evisceration.^[11,12]^ It was predicted that 610,821 eyes will go blind each year after FK infection.^[12]^

The most common causative organisms of FK are *Fusarium*, *Aspergillus*, and *Candida* species, which account for more than 95% of reported cases.^[12,13]^ The specific fungal species that cause FK are related to the unique risk factors. Ocular trauma or corneal abrasion sustained during agricultural activities is an important risk factor for filamentous fungal infection.^[14,15]^ FK caused by *Candida* spp. is more common in immunocompromised patients or patients with the ocular surface disease and a history of topical steroid use.^[16,17]^ Keratorefractive surgery (such as laser *in situ* keratomileusis) has been associated with increased risk for FK. Isolated organisms included *Fusarium*, *Aspergillus*, *Alternaria*, and *Candida* species.^[18–21]^

Simultaneous development of fungal infections in both eyes after ptosis surgery is highly unusual. In addition, *T asahii* rarely causes FK. *Trichosporon* is a yeast-like fungus, and some *Trichosporon* species constitute a natural part of the microbiota of human skin.^[5]^ This organism can be involved in a variety of conditions, from a harmless skin hair condition and white piedra to a serious, life-threatening opportunistic infection in immunocompromised patients.^[22,23]^

Known risk factors for FK are trauma, contact lens use, topical medications (corticosteroids), corneal surgery, chronic keratitis, and immunosuppression.^[5,24,25]^ The patient in this case report had multiple risk factors for FK. First, the ptosis surgery could have led to corneal exposure. Although we observed complete lid closure and no corneal exposure in the clinic, the patient's family members reported that her eyelids were only partially closed while she slept. We suspect that this patient's partial lid opening led to her exposure to keratitis and the development of epithelial defects. Second, the persistent and refractory epithelial defects made the cornea vulnerable to further microbial infection. A breach of the corneal epithelium is the most common risk factor for the development of FK.^[26]^ Third, steroid eye drop use after amniotic membrane transplantation induced local immunosuppression, which made infection more likely. Lastly, a previous report suggested contact lens wear as a risk factor for *T asahii* keratitis;^[5]^ therefore, the therapeutic contact lenses worn by our patient following ptosis surgery and the amniotic membrane transplantation might have led to the aggravation of the corneal ulcers.

When 0.15% amphotericin B was used hourly for an initial 3-week postoperative period, clinical improvement was nonexistent, and the epithelial defects persisted. We speculated that lid revision surgery to release the levator muscle adhesion would lessen the corneal exposure and alter the corneal microenvironment to be more favorable for healing. We also changed the topical antifungal agent from 0.15% amphotericin B to 1% voriconazole. Recent studies have shown that patients with *Trichosporon* fungemia had a lower mortality rate when they received voriconazole treatment.^[27,28]^ In addition, voriconazole showed the highest antifungal activity in vitro against *Trichosporon* isolates.^[28]^

To the best of authors’ knowledge, this is the first case report of a bilateral case of *Trichosporon* FK that occurred following ptosis correction surgery. It will be necessary to identify the specific risk factors and mediate them when possible to save the cornea from serious complications of FK.

## Author contributions

**Conceptualization:** Jeongah Shin, Kyong Jin Cho, Chang Rae Rho.

**Data curation:** Jeongah Shin, Chang Rae Rho.

**Formal analysis:** Kyong Jin Cho.

**Methodology:** Woo Young Son, Kyong Jin Cho.

**Project administration:** Chang Rae Rho.

**Supervision:** Kyong Jin Cho, Chang Rae Rho.

**Validation:** Chang Rae Rho.

**Visualization:** Chang Rae Rho.

**Writing – original draft:** Jeongah Shin, Chang Rae Rho.

**Writing – review & editing:** Woo Young Son, Chang Rae Rho.
